# Airborne pollen concentrations and daily mortality from respiratory and cardiovascular causes

**DOI:** 10.1093/eurpub/ckab034

**Published:** 2021-04-05

**Authors:** Jouni J K Jaakkola, Simo-Pekka Kiihamäki, Simo Näyhä, Niilo R I Ryti, Timo T Hugg, Maritta S Jaakkola

**Affiliations:** 1 Center for Environmental and Respiratory Health Research, University of Oulu, Oulu, Finland; 2 Biocenter Oulu, University of Oulu, Oulu, Finland; 3 Medical Research Center Oulu, University Hospital and University of Oulu, Oulu, Finland; 4 Finnish Meteorological Institute, Helsinki, Finland

## Abstract

We conducted a time-series analysis of the relations between daily levels of allergenic pollen and mortality in the Helsinki Metropolitan Area with 153 378 deaths; 9742 from respiratory and 57 402 from cardiovascular causes. Daily (average) pollen counts of alder, birch, mugwort and grass were measured. In quasi-Poisson regression analysis, abundant alder pollen increased the risk of non-accidental deaths with an adjusted cumulative mortality rate ratio (acMRR) of 1.10 (95% CI 1.01–1.19) and of deaths from respiratory-diseases with acMRR of 1.78 (95% CI 1.19–2.65). Abundant mugwort pollen increased cardiovascular mortality (1.41, 1.02–1.95). These findings identify an important global public health problem.

## Introduction

Exposures to pollen from certain trees, grasses and herbs have been associated with some symptoms and signs of allergic reactions among subjects with asthma and allergies.^1^ These effects are linked to type I allergic sensitization, which is mediated by specific IgE antibodies in the eyes and upper and lower airways.^2^ Daily variation of pollen associates with exacerbations of asthma, and with daily outpatient visits and hospitalizations attributable to asthma and chronic obstructive pulmonary disease (COPD).^1^^,^^3^^,^^4^

We identified only one previous study that had assessed the relation between airborne pollen concentrations and daily respiratory and cardiovascular mortality. Brunekreef and colleagues^5^ studied the relations of cardiovascular and respiratory mortality to the levels of most frequently occurring pollen, including grass, birch and oak pollen, in the Netherlands. In addition, respiratory and cardiovascular mortality was related to ash (*Fraxinus*), mugwort (*Artemisia*) and dock (*Rumex*) pollen. In Poisson regression analysis, a dose-response relation was present between the weekly average level of grass pollen and mortality from cardiovascular disease, COPD and pneumonia.

We tested the hypothesis that daily exposure to the most common allergenic pollens in Finland, including alder (*Alnus*), birch (*Betula)*, grass (*Poaceae*) and mugwort increases respiratory and cardiovascular mortality.

## Methods

### Mortality data

We conducted a time-series analysis of potential relations between daily levels of pollen and mortality from respiratory, cardiovascular and all non-violent causes in the Helsinki Metropolitan Area, Finland, in 1994–2014. The data provided by the Statistics Finland comprised 153 378 deaths, of which 9742 (6.4%) were from respiratory (ICD-9 460–519; ICD-10 J00–J99), 57 402 (37.4%) from cardiovascular (ICD-9 390–459; ICD-10 I00–I99) and 139 540 from non-accidental causes (ICD-9 001–799; ICD-10 A00–R99).

### Pollen monitoring

Daily pollen counts of alder, birch, grass and mugwort were obtained from a regional pollen monitoring station located on the roof of the Helsinki University Hospital, Skin and Allergy Hospital being centrally in the Helsinki Metropolitan Area (surface area 772.3 km^2^). Burkard 7 day recording volumetric trap was used in pollen sampling. Sampling methods and data interpretation followed the standard methodology of the European Aeroallergen Network (http://www.polleninfo.org/). Pollen data included the periods March–August each year. Air quality data, including daily concentrations of PM_2.5_, SO_2_ and O_3_, was from the City of Helsinki Environment Office and daily temperatures from the Finnish Meteorological Institute. The exposure parameters of interest were the mean daily number of pollen grains per m^3^ of air for each pollen of interest.

### Statistical methods

The distribution of death counts deviated significantly from the Poisson distribution, when tested by likelihood ratio test. Therefore, we used quasi-Poisson regression analysis to estimate mortality rate ratios (MRR) related to pollen counts. We followed the analytical approach presented by Bashkaran and colleagues.^6^ First, we fitted models containing each pollen concentration as the only explanatory variable. Pollen counts were treated as categorical variables in *a priori* exposure categories: low (<10 grains/m^3^), moderate (10–100 grains/m^3^ for alder and birch and 10–30 grains/m^3^ for grasses and mugwort) and abundant (>100 grains/m^3^ for alder and birch and >30 grains/m^3^ for grasses and mugwort), based on the Finnish national pollen monitoring centres.^7^ We adjusted cumulative mortality rate ratios (acMRR) for temporal variation applying cubic splines (4 df/year), for PM_2.5_, SO_2_ and O_3_, and for *dlnm* models for mean daily temperature up to 21 lag days.^6^ We conducted sensitivity analyses fitting different time lag structures for pollen, temperature and air pollutants.

## Results

The median numbers of daily deaths were 20 (interquartile range 17–23) for all causes, 18 (IQR 15–21) for non-accidental, 7 (IQR 6–9) for cardiovascular and 1 (IQR 0–2) for respiratory-diseases. In quasi-Poisson regression analysis adjusting for confounding, abundant alder pollen increased the risk of non-accidental deaths with acMRR of 1.10 (95% Confidence interval 1.01–1.19), deaths from respiratory-diseases with acMRR of 1.78 (1.19–2.65). Moderate birch pollen (1.37, 1.02–1.85), but not abundant (0.976, 0.753–1.265), increased mortality from respiratory causes (table 1). Abundant mugwort pollen increased the risk of cardiovascular deaths with acMRR of 1.41 (95% CI 1.02–1.95).

**Table 1:** Adjusted cumulative mortality rate ratios (MRRs) and their 95% confidence intervals (CI) associated with 7–14 day lag concentrations of most frequently occurring airborne pollen in the Helsinki Metropolitan Area, 1994–2014

	Lag	Pollen exposure concentration		
		Low^a^	Moderate^b^	Abundant^c^
		Reference	Adjusted MRR^d^ (95% CI)	Adjusted MRR^d^ (95% CI)
Alder				
Respiratory disease	14	1.000	0.847 (0.614–1.172)	1.777 (1.191–2.651)
Cardiovascular disease	7	1.000	1.037 (0.935–1.150)	1.044 (0.918–1.187)
Non-accidental causes	7	1.000	0.984 (0.920–1.052)	1.095 (1.008–1.189)
Birch				
Respiratory disease	14	1.000	1.370 (1.017–1.846)	0.976 (0.753–1.265)
Cardiovascular disease	7	1.000	0.969 (0.879–1.069)	0.977 (0.890–1.072)
Non-accidental causes	7	1.000	1.017 (0.956–1.083)	0.968 (0.912–1.028)
Grasses				
Respiratory disease	14	1.000	1.117 (0.774–1.612)	1.299 (0.879–1.920)
Cardiovascular disease	7	1.000	1.010 (0.911–1.119)	1.047 (0.930–1.179)
Non-accidental causes	7	1.000	1.033 (0.967–1.103)	1.065 (0.986–1.151)
Mugwort				
Respiratory disease	14	1.000	1.246 (0.752–2.064)	1.322 (0.441–3.964)
Cardiovascular disease	10	1.000	0.954 (0.808–1.127)	1.414 (1.024–1.953)
Non-accidental causes	10	1.000	0.957 (0.859–1.065)	1.191 (0.961–1.475)

a Low = Less than 10 grains/m^3^.

b Moderate = 10–100 grains/m^3^ for alders and birches and 10–30 grains/m^3^ for grasses and mugwort.

c Abundant = Over 100 grains/m^3^ for alders and birches and over 30 grains/m^3^ for grasses and mugwort.

d Quasi-Poisson regression: Cumulative MRRs calculated from 7 to 14 lag day dlnm models adjusting for temporal variations and index day PM_2.5_ concentration, 3-day lagged SO_2_ concentration and temperature dlnm.

## Discussion

Our findings are consistent with the hypothesis that exposure to abundant levels of alder pollen increases significantly non-accidental mortality and the risk of death from respiratory causes and exposure to abundant levels of mugwort pollen increases significantly cardiovascular mortality. There was also some suggestive evidence that exposure to grass pollen increases respiratory and cardiovascular mortality, which is in line with the previous Dutch study.^5^ The findings on birch pollen effect are inconsistent. A Canadian case-crossover study^8^ showed recently an association between daily mean aeroallergen concentrations (pollen and moulds) and the amount of emergency room visits for myocardial infarction, in line with present findings on cardiovascular mortality. The risk in their study was 5.5% higher (95% CI 3.4–7.6%) during days of the highest aeroallergen exposure tertile compared to the lowest.

Alder pollen season takes place in March–April in Finland, and thus, it is the first major plant group pollinating in the spring. It is followed by birch, the most important allergenic plant in the region. There is limited knowledge about the role of alder pollen in allergic reactions. Based on a report from a Global Asthma and Allergy European Network study,^9^ the sensitization rates for alder are 26.3% (19.5–33.2%) in Finland and 21.2% (95% CI 19.8–22.7%) in Europe in general. In a Japanese study reported by Maeda et al.,^10^ where allergic reactions were measured using Radioallergosorbent Test, the reactions to alder and birch were almost identical, indicating substantial cross-reactivity between alder and birch pollen. Surprisingly, mortality rates in the present study were clearly related to the levels of alder pollen, whereas no consistent relation was observed with birch pollen levels. It can be speculated that alders as the first sources of allergen load could prime the immunologic system of allergic people. Therefore, exposure to similar kind of allergens as alder may provoke immunologic system, elicit first adverse reactions producing an ‘immunologic shock’ and therefore, harvest susceptible individuals already before the birch flowering takes place. Our study did not involve measurements, which would elaborate potential biological mechanisms how exposure to pollen could increase the risk of death from respiratory and cardiovascular causes. However, it is not unusual in the tradition of epidemiological research that the first observation about an association between environmental exposure and the risk of disease is made at a point of time when there is little understanding about potential biological mechanisms. Such empirical observations should not be refuted, on the contrary, novel findings should be presented to the scientific community, so that similar findings can be either repeated or refuted in future studies.

In conclusion, in this study we provide new evidence that increased pollen levels from alder trees increase significantly mortality from non-accidental and respiratory causes and mugwort pollen increases cardiovascular mortality. These findings suggest that exposure to pollen may be a more serious public health problem than previously understood. Thus, subjects with sensitivity to alder should get appropriate medication during alder season as well as advice to avoid areas with high levels of alder pollen. The impact of different pollen exposures in different climatic zones needs further elaboration.

## Funding

This study was supported by the Academy of Finland [grant numbers 266314 (APTA Consortium) and 310372 (GLORIA Consortium)] and The Research Foundation of the Pulmonary Diseases. The funders had no role in study design, data collection or analysis, decision to publish, or preparation of the manuscript. 

*Conflicts of interest*: None declared.

## Ethics approval

The study protocol was approved by the Ethics Committee of the Statistics Finland.

**Key points**

- Exposure to pollen from certain trees, grasses and herbs have been associated with a range of symptoms and signs of allergic reactions among subjects with asthma and allergies.
- This study conducted in the Helsinki Metropolitan Ares provides new evidence that increased pollen levels from alder trees increase significantly mortality from non-accidental and respiratory causes and exposure to mugwort pollen increases cardiovascular mortality.
- These findings identify an important global public health impact and the results call for development of population-based prevention through alarm systems and personalized medical advice at the clinical practices.
